# Supplementary material for: A computational model tracks whole-lung Mycobacterium tuberculosis infection and predicts factors that inhibit dissemination
Source: PLoS Comput Biol. 2020 May 20;16(5):e1007280. doi: 10.1371/journal.pcbi.1007280 (PMC7239387; doi:10.1371/journal.pcbi.1007280)
Supplement: S1 Equations — (PDF) [file pcbi.1007280.s001.pdf]

1 Granuloma Ordinary Differential Equations

*Note: in these equations m is the same as $CD8MultiFunc$ in the text. m is defined as $m = \dfrac{frac + 1}{2}$, where $frac$ is the fraction of all new CD8 cells that are multifunctional.

$$
\begin{aligned}
\frac{dB_E}{dt} = & \\
& + \alpha_{20} B_E \text{ (BE replication)} \\
& + k_{17} N M_I \left(\frac{B_I^2}{B_I^2 + N^2 M_I^2} \right) \text{ (MI bursting)} \\
& + k_{14a} N_{fracc} \frac{B_I}{MI} M_I \left(\frac{\left(\frac{T_C + w_3 T_1}{M_I} \right)}{\left(\frac{T_C + w_3 T_1}{M_I} \right) + c_4} \right) \text{ (Tcell-induced MI apoptosis)} \\
& + k_{14b} N_{fraca} \frac{B_I}{MI} M_I \left(\frac{F_\alpha}{F_\alpha + f_9 I_{10} + s_{4b}} \right) \text{ (TNF-}\alpha\text{-induced MI apoptosis)} \\
& - k_2 \frac{N}{2} M_R \left(\frac{B_E}{B_E + c_9} \right) \text{ (MR internalization)} \\
& - k_{15} M_A B_E \text{ (MA killing)} \\
& - k_{18} M_R B_E \text{ (MR killing)} \\
& - \mu_{B_E} B_E \text{ (BE natural death)} \\
& + \mu_{M_I} N_{fracd} \frac{B_I}{MI} M_I \text{ (MI natural death)}
\end{aligned}
$$

$$\frac{dB_I}{dt} =$$

$$+\alpha_{19} B_I \left(NMI - B_I\right) \text{ (BI replication)}$$

$$+k_2 \frac{N}{2} M_R \left(\frac{B_E}{B_E + c_9}\right) \text{ (MR internalization)}$$

$$-k_{17} N M_I \left(\frac{B_I^2}{B_I^2 + N^2 M_I^2}\right) \text{ (MI bursting)}$$

$$-k_{14a} \frac{B_I}{MI} M_I \left(\frac{\left(\frac{T_C + w_3 T_1}{M_I}\right)}{\left(\frac{T_C + w_3 T_1}{M_I}\right) + c_4}\right) \text{ (Tcell-induced MI apoptosis)}$$

$$-k_{14b} \frac{B_I}{MI} M_I \left(\frac{F_\alpha}{F_\alpha + f_9 I_{10} + s_{4b}}\right) \text{ (TNF-}\alpha\text{-induced MI apoptosis)}$$

$$-k_{52} \frac{B_I}{MI} M_I \left(\frac{\left(\frac{T_C \left(\frac{T_1}{T_1 + c_{T_1}}\right) + w_1 T_1}{M_I}\right)}{\left(\frac{T_C \left(\frac{T_1}{T_1 + c_{T_1}}\right) + w_1 T_1}{M_I}\right) + c_{52}}\right) \text{ (Tcell cytotoxic MI killing)}$$

$$-\mu_{B_I} B_I \text{ (BI death inside living MI)}$$

$$-\mu_{M_I} \frac{B_I}{MI} M_I \text{ (MI natural death)}$$

$$\frac{dM_R}{dt} =$$

$$+Sr_M \text{ (Background recruitment)}$$

$$+\alpha_{4a}(M_A + w_2 M_I) \text{ (MA/MI recruitment)}$$

$$+Sr_{4b} \left(\frac{F_\alpha}{F_\alpha + f_8 I_{10} + s_{4b}}\right) \text{ (TNF-}\alpha\text{ recruitment)}$$

$$-k_2 M_R \left(\frac{B_E}{B_E + c_9}\right) \text{ (MR infection)}$$

$$-k_3 M_R \left(\frac{B_E + wB_I + \beta F_\alpha}{B_E + wB_I + \beta F_\alpha + c_8}\right) \left(\frac{I_\gamma}{I_\gamma + f_1 I_4 + f_7 I_{10} + s_1}\right) \text{ (MR activation)}$$

$$-\mu_{M_R} M_R \text{ (MR natural death)}$$

$$\frac{dM_I}{dt} =$$

$$+ k_2 M_R \left(\frac{B_E}{B_E + c_9}\right) \text{ (MR infection)}$$

$$- k_{17} M_I \left(\frac{B_I^2}{B_I^2 + N^2 M_I^2}\right) \text{ (MI Bursting)}$$

$$- k_{14a} M_I \left(\frac{\left(\frac{T_C + w_3 T_1}{M_I}\right)}{\left(\frac{T_C + w_3 T_1}{M_I}\right) + c_4}\right) \text{ (Tcell-induced MI apoptosis)}$$

$$- k_{14b} M_I \left(\frac{F_\alpha}{F_\alpha + f_9 I_{10} + s_{4b}}\right) \text{ (TNF-}\alpha\text{-induced MI apoptosis)}$$

$$- k_{52} M_I \left(\frac{\left(\frac{T_C\left(\frac{T_1}{T_1 + c_{T_1}}\right) + w_1 T_1}{M_I}\right)}{\left(\frac{T_C\left(\frac{T_1}{T_1 + c_{T_1}}\right) + w_1 T_1}{M_I}\right) + c_{52}}\right) \text{ (Tcell cytotoxic MI killing)}$$

$$- \mu_{M_I} M_I \text{ (MI natural death)}$$

$$\frac{dM_A}{dt} =$$

$$+ k_3 M_R \left(\frac{B_E + wB_I + \beta F_\alpha}{B_E + wB_I + \beta F_\alpha + c_8}\right) \left(\frac{I_\gamma}{I_\gamma + f_1 I_4 + f_7 I_{10} + s_1}\right) \text{ (MA activation)}$$

$$- k_4 M_A \left(\frac{I_{10}}{I_{10} + s_8}\right) \text{ (MA deactivation)}$$

$$- \mu_{M_A} M_A \text{ (MA natural death)}$$

$$\frac{dT_0}{dt} =$$

$$+ \alpha_{1a} (M_A + w_2 M_I) \text{ (Mac T0 recruitment)}$$

$$+ Sr_{1b} \left(\frac{F_\alpha}{F_\alpha + f_8 I_{10} + s_{4b2}} \right) \text{ (TNF-}\alpha\text{ recruitment)}$$

$$+ \alpha_2 T_0 \left(\frac{M_A}{M_A + c_{15}} \right) \text{ (T0 proliferation)}$$

$$- k_6 I_{12} T_0 \left(\frac{I_\gamma}{I_\gamma + (f_1 I_4 + f_7 I_{10}) + s_1} \right) \text{ (T0 to Th1 differentiation)}$$

$$- k_7 T_0 \left(\frac{I_4}{I_4 + f_2 I_\gamma + s_2} \right) \text{ (T0 to Th2 differentiation)}$$

$$- \mu_{T_0} T_0 \text{ (T0 natural death)}$$

$$\frac{dT_1}{dt} =$$

$$+ \alpha_{3a} (M_A + w_2 M_I) \text{ (Mac Th1 recruitment)}$$

$$+ Sr_{3b} \left(\frac{F_\alpha}{F_\alpha + f_8 I_{10} + s_{4b1}} \right) \text{ (TNF-}\alpha\text{ recruitment)}$$

$$+ k_6 I_{12} T_0 \left(\frac{I_\gamma}{I_\gamma + (f_1 I_4 + f_7 I_{10}) + s_1} \right) \text{ (T0 to Th1 differentiation)}$$

$$- \mu_{T_\gamma} \left(\frac{I_\gamma}{I_\gamma + c} \right) T_1 M_A \text{ (IFN-}\alpha\text{ Th1 apoptosis)}$$

$$- \mu_{T_1} T_1 \text{ (Th1 natural death)}$$

$$\frac{dT_2}{dt} =$$

$$+ \alpha_{3a2} (M_A + w_2 M_I) \text{ (Mac Th2 recruitment)}$$

$$+ Sr_{3b2} \left(\frac{F_\alpha}{F_\alpha + f_8 I_{10} + s_{4b1}} \right) \text{ (TNF-}\alpha\text{ Th2 recruitment)}$$

$$+ k_7 T_0 \left(\frac{I_4}{I_4 + f_2 I_\gamma + s_2} \right) \text{ (T0 to Th2 differentiation)}$$

$$- \mu_{T_2} T_2 \text{ (Th2 natural death)}$$

$$\frac{dT_{80}}{dt} =$$

$$+ \alpha_{1a} \left(M_A + w_2 M_I\right) \text{ (Mac T80 recruitment)}$$

$$+ Sr_{1b} \left(\frac{F_\alpha}{F_\alpha + f_8 I_{10} + s_{4b2}}\right) \text{ (TNF-}\alpha\text{ T80 recruitment)}$$

$$+ \alpha_2 T_{80} \left(\frac{M_A}{M_A + c_{15}}\right) \text{ (T80 proliferation)}$$

$$- k_6 I_{12} T_{80} \left(\frac{I_\gamma}{I_\gamma + (f_1 I_4 + f_7 I_{10}) + s_1}\right) \text{ (T80 to TC/T8 differentiation)}$$

$$- \mu_{T_0} T_{80} \text{ (T80 natural death)}$$

$$\frac{dT_8}{dt} =$$

$$+ m\alpha_{3ac} \left(M_A + w_2 M_I\right) \text{ (Mac T8 recruitment)}$$

$$+ mSr_{3bc} \left(\frac{F_\alpha}{F_\alpha + f_8 I_{10} + s_{4b1}}\right) \text{ (TNF-}\alpha\text{ T8 recruitment)}$$

$$+ mk_6 I_{12} T_{80} \left(\frac{I_\gamma}{I_\gamma + (f_1 I_4 + f_7 I_{10}) + s_1}\right) \text{ (T80 to TC/T8 differentiation)}$$

$$- \mu_{T_{c\gamma}} \left(\frac{I_\gamma}{I_\gamma + c_c}\right) T_8 M_A \text{ (IFN-}\alpha\text{ T8 apoptosis)}$$

$$- \mu_{T_C} T_8 \text{ (T8 natural death)}$$

$$\frac{dT_C}{dt} =$$

$$+ m\alpha_{3ac} \left(M_A + w_2 M_I\right) \text{ (Mac TC recruitment)}$$

$$+ mSr_{3bc} \left(\frac{F_\alpha}{F_\alpha + f_8 I_{10} + s_{4b1}}\right) \text{ (TNF-}\alpha\text{ TC recruitment)}$$

$$+ mk_6 I_{12} T_{80} \left(\frac{I_\gamma}{I_\gamma + (f_1 I_4 + f_7 I_{10}) + s_1}\right) \text{ (T80 to TC/T8 differentiation)}$$

$$- \mu_{T_{c\gamma}} \left(\frac{I_\gamma}{I_\gamma + c_c}\right) T_C M_A \text{ (IFN-}\alpha\text{ TC apoptosis)}$$

$$- \mu_{T_C} T_C \text{ (TC natural death)}$$

$$\frac{dF_\alpha}{dt} =$$

$$+\alpha_{30}M_I \text{ (MI TNF-}\alpha\text{ production)}$$

$$+\alpha_{31}M_A\left(\frac{I_\gamma+\beta_2(B_E+wB_I)}{I_\gamma+\beta_2(B_E+wB_I)+f_1I_4+f_7I_{10}+s_{10}}\right) \text{ (MA TNF-}\alpha\text{ production)}$$

$$+\alpha_{32}T_1 \text{ (Th1 TNF-}\alpha\text{ production)}$$

$$+\alpha_{33}\left(\frac{T_C+T_8}{2m}\right) \text{ (CD8+ TNF-}\alpha\text{ production)}$$

$$-\mu_{F_\alpha}F_\alpha \text{ (TNF-}\alpha\text{ natural decay)}$$

$$\frac{dI_\gamma}{dt} =$$

$$+s_g\left(\frac{B_E+wB_I}{B_E+wB_I+c_{10}}\right)\left(\frac{I_{12}}{I_{12}+s_7}\right) \text{ (DC IG production)}$$

$$+\alpha_{5a}T_1\left(\frac{M_A}{M_A+c_{5a}}\right) \text{ (Th1 IG production)}$$

$$+\alpha_{5b}T_8\left(\frac{M_A}{M_A+c_{5b}}\right) \text{ (T8 IG production)}$$

$$+\alpha_{5c}M_I \text{ (MI IG production)}$$

$$+\alpha_7T_0\left(\frac{I_{12}}{I_{12}+f_4I_{10}+s_4}\right) \text{ (T0 IG production)}$$

$$+\alpha_7T_{80}\left(\frac{I_{12}}{I_{12}+f_4I_{10}+s_4}\right) \text{ (T80 IG production)}$$

$$-\mu_{I_\gamma}I_\gamma \text{ (IG natural decay)}$$

$$\frac{dI_{12}}{dt} =$$

$$+s_{12}\left(\frac{B_E+wB_I}{B_E+wB_I+c_{230}}\right) \text{ (DC I12 production)}$$

$$+\alpha_{23}M_R\left(\frac{B_E+wB_I}{B_E+wB_I+c_{23}}\right) \text{ (MR I12 production)}$$

$$+\alpha_8M_A\left(\frac{s}{s+I_{10}}\right) \text{ (MA I12 production)}$$

$$-\mu_{I_{12}}I_{12} \text{ (I12 natural decay)}$$

$$
\begin{aligned}
\frac{dI_{10}}{dt} = & \\
& + \delta_7 \left(M_I + M_A\right) \left(\frac{s_6}{I_{10} + f_6 I_\gamma + s_6} \right) \text{ (MI I10 production)} \\
& + \alpha_{16} T_1 \text{ (Th1 I10 production)} \\
& + \alpha_{17} T_2 \text{ (Th2 I10 production)} \\
& + \alpha_{18} \left(\frac{T_C + T_8}{2m} \right) \text{ (CD8 I10 production)} \\
& - \mu_{I_{10}} I_{10} \text{ (I10 natural decay)}
\end{aligned}
$$

$$
\begin{aligned}
\frac{dI_4}{dt} = & \\
& + \alpha_{11} T_0 \text{ (T0 I4 production)} \\
& + \alpha_{12} T_2 \text{ (T2 I4 production)} \\
& - \mu_{I_4} I_4 \text{ (I4 natural decay)}
\end{aligned}
$$

2 Dissemination Equations

2.1 Local Dissemination

2.1.1 Probability

$$Prob_{Local}(t) = \lambda_{Local} \frac{CFU(t)}{CFU(t) + CFU_{half}^{Local}}$$

2.1.2 Expected Value

$$E_{Local}(t+dt) = E_{Local}(t) + \int_t^{dt} Prob_{Local}(s) ds \approx E_{Local}(t) + \lambda_{Local} \frac{CFU(t)}{CFU(t) + CFU_{half}^{Local}} dt$$

2.2 Number of Dissemination Events

$NumDiss_{Local}(t + dt) = Floor\left(Res_{Local}(t) + E_{Local}(t + dt) - E_{Local}(t)\right)$
$Res_{Local}(t+dt) = (Res_{Local}(t) + E_{Local}(t + dt) - E_{Local}(t)) - NumDiss_{Local}(t+dt)$

2.2.1 Updated Counts/Concentrations

$$MI^{Parent} = \begin{cases} MI^{Parent} - 1 & \text{if } MI^{Parent} \geq 1 \\ 0 & \text{if } MI^{Parent} < 1 \end{cases}$$

$$BI^{Parent} = \begin{cases} BI^{Parent} - \dfrac{BI^{Parent}}{MI^{Parent}} & \text{if } MI^{Parent} \geq 1 \\ 0 & \text{if } MI^{Parent} < 1 \end{cases}$$

$T_0^{Parent,New}(t) = T_0^{Parent,Old}(t) - Norm(\mu_{Local}^{Tfrac}, \sigma_{Local}^{Tfrac}) * T_0^{Parent,Old}(t)$
$T_1^{Parent,New}(t) = T_1^{Parent,Old}(t) - Norm(\mu_{Local}^{Tfrac}, \sigma_{Local}^{Tfrac}) * T_1^{Parent,Old}(t)$
$T_2^{Parent,New}(t) = T_2^{Parent,Old}(t) - Norm(\mu_{Local}^{Tfrac}, \sigma_{Local}^{Tfrac}) * T_2^{Parent,Old}(t)$
$T_{80}^{Parent,New}(t) = T_{80}^{Parent,Old}(t) - Norm(\mu_{Local}^{Tfrac}, \sigma_{Local}^{Tfrac}) * T_{80}^{Parent,Old}(t)$
$T_C^{Parent,New}(t) = T_C^{Parent,Old}(t) - Norm(\mu_{Local}^{Tfrac}, \sigma_{Local}^{Tfrac}) * T_C^{Parent,Old}(t)$
$T_8^{Parent,New}(t) = T_8^{Parent,Old}(t) - Norm(\mu_{Local}^{Tfrac}, \sigma_{Local}^{Tfrac}) * T_8^{Parent,Old}(t)$
Other counts/concentrations remain unchanged

$$MI^{Daughter} = \begin{cases} 1 & \text{if } MI^{Parent} \geq 1 \\ MI^{Parent} & \text{if } MI^{Parent} < 1 \end{cases}$$

$$BI^{Daughter} = \begin{cases} \dfrac{BI^{Parent}}{MI^{Parent}} & \text{if } MI^{Parent} \geq 1 \\ BI^{Parent} & \text{if } MI^{Parent} < 1 \end{cases}$$

$T_0^{Daughter}(t) = Norm(\mu_{Local}^{Tfrac}, \sigma_{Local}^{Tfrac}) * T_0^{Parent,Old}(t)$
$T_1^{Daughter}(t) = Norm(\mu_{Local}^{Tfrac}, \sigma_{Local}^{Tfrac}) * T_1^{Parent,Old}(t)$
$T_2^{Daughter}(t) = Norm(\mu_{Local}^{Tfrac}, \sigma_{Local}^{Tfrac}) * T_2^{Parent,Old}(t)$
$T_{80}^{Daughter}(t) = Norm(\mu_{Local}^{Tfrac}, \sigma_{Local}^{Tfrac}) * T_{80}^{Parent,Old}(t)$
$T_C^{Daughter}(t) = Norm(\mu_{Local}^{Tfrac}, \sigma_{Local}^{Tfrac}) * T_C^{Parent,Old}(t)$
$T_8^{Daughter}(t) = Norm(\mu_{Local}^{Tfrac}, \sigma_{Local}^{Tfrac}) * T_8^{Parent,Old}(t)$
Other counts/concentrations are 0

2.2.2 Location

$x_{Daughter} = x_{Parent} + R_{rand} * \cos(\theta_{rand}) * \sin(\phi_{rand})$
$y_{Daughter} = y_{Parent} + R_{rand} * \sin(\theta_{rand}) * \sin(\phi_{rand})$
$z_{Daughter} = z_{Parent} + R_{rand} * \cos(\phi_{rand})$
where:
$R_{rand} = Norm(\mu_{Local}^{Dist}, \sigma_{Local}^{Dist})$
$\theta_{rand} = Unif(0,1) * 2 * \pi$
$\phi_{rand} = Unif(0,1) * \pi$
and:
$(x_{Daughter}, y_{Daughter}, z_{Daughter}) \in \Gamma$

2.3 Nonocal Dissemination

2.3.1 Probability

$$Prob_{Nonlocal}(t) = \lambda_{Nonlocal}\frac{CFU(t)}{CFU(t) + CFU_{half}^{Nonlocal}}$$

2.3.2 Expected Value

$$E_{Nonlocal}(t{+}dt) = E_{Nonlocal}(t){+}\int_t^{dt} Prob_{Local}(s)ds \approx E_{Nonlocal}(t){+}\lambda_{Nonlocal}\frac{CFU(t)}{CFU(t) + CFU_{half}^{Nonlocal}}dt$$

2.4 Number of Dissemination Events

$NumDiss_{Nonlocal}(t{+}dt) = Floor\left(Res_{Nonlocal}(t) + E_{Nonlocal}(t + dt) - E_{Nonlocal}(t)\right)$
$Res_{Nonlocal}(t{+}dt) = \left(Res_{Nonlocal}(t) + E_{Nonlocal}(t + dt) - E_{Nonlocal}(t)\right) - NumDiss_{Nonlocal}(t+dt)$

2.4.1 Updated Counts/Concentrations

$BE^{Parent} = BE^{Parent} - 1$
Other counts/concentrations remain unchanged

$MI^{Daughter} = 1$
$BI^{Daughter} = 1$
Other counts/concentrations are 0

2.4.2 Location

$x_{Daughter} = x_{min} + Unif(0, 1) * (x_{max} - x_{min})$
$y_{Daughter} = y_{min} + Unif(0, 1) * (y_{max} - y_{min})$
$z_{Daughter} = z_{min} + Unif(0, 1) * (z_{max} - z_{min})$
where:

$(x_{Daughter}, y_{Daughter}, z_{Daughter}) \in \Gamma$
