# Supplementary material for: A computational model tracks whole-lung Mycobacterium tuberculosis infection and predicts factors that inhibit dissemination
Source: PLoS Comput Biol. 2020 May 20;16(5):e1007280. doi: 10.1371/journal.pcbi.1007280 (PMC7239387; doi:10.1371/journal.pcbi.1007280)
Supplement: S1 Table — (DOCX) [file pcbi.1007280.s002.docx]

| **Parameter name** | **Min** | **Max** | **Units** | **Ref** | **Description** |
| --- | --- | --- | --- | --- | --- |
| Srm | 0 | 0 | 1/day | fit | MR recruitment rate |
| alpha4a | 0.57 | 0.83 | 1/day | [1], [2], fit | Macrophage recruitment of MR |
| w | 0.29 | 0.33 | -- | [3], [2], fit | Contribution of BI to MR activation |
| w3 | 0.23 | 0.37 | -- | [1], [2], fit | Max contribution of Th1 to MI apoptosis |
| w2 | 1 | 1 | -- | [3], [2], fit | Contribution of MI to MR recruitment |
| Sr4b | 650 | 750 | 1/day | [1], [2], fit | Falpha-dependent recruitment of MR |
| f8 | 0.002 | 0.002 | -- | fit | Ratio adjustment IL-10/Falpha on MR recruitment |
| f9 | 0.6 | 0.6 | -- | fit | Ratio adjustment Falpha/IL-10 |
| s4b | 3210 | 4860 | pg/ml | [4], [2], fit | Half saturation of Falpha on MR recruitment |
| s4b1 | 6780 | 9410 | pg/ml | [1], [2], fit | Half saturation of Falpha dependent Th1 recruitment |
| s4b2 | 5340 | 9420 | pg/ml | [4], [2], fit | Half saturation of Falpha-dependent T0 recruitment |
| k4 | 0.074 | 0.17 | 1/day | [3], [2], fit | MA deactivation by IL-10 |
| s8 | 200 | 940 | pg/ml | [3], [2], fit | Half saturation of IL-10 on MA deactivation |
| k2 | 0.43 | 2.2 | 1/day | [3], [2], fit | MR infection rate |
| c9 | 1190 | 7450 | count | [3], [2], fit | Half saturation of BE on MR infection |
| k3 | 0.04 | 0.04 | 1/day | [3], [2], fit | MR activation rate |
| f1 | 150 | 150 | -- | [3], [2], fit | Adjustment IL-4/IFNg |
| s1 | 54 | 450 | pg/ml | [3], [2], fit | Half saturation of IFNg-dependent MR activation |
| Beta | 1.00E+07 | 1.00E+07 | 1/pg | [1], [2], fit | Scaling factor of Falpha for MR activation |
| c8 | 175370 | 363170 | count | fit | Half saturation of BE and BI on MR activation |
| nuMR | 0.005 | 0.005 | 1/day | [3], [2], fit | MR death rate |
| k17 | 0.1 | 0.3 | 1/day | [3], [2], fit | Max rate of MI bursting |
| N | 20 | 25 | count | [3], [2], [5], fit | Carrying capacity of MI |
| k14a | 0.06 | 0.34 | 1/day | [1], [2], fit | T cell induced apoptosis of MI |
| c4 | 400 | 880 | -- | [3], [2], fit | Half saturation of Th1/MI ratio on MI apoptosis |
| k14b | 0.63 | 0.86 | 1/day | [1], [2], fit | Falpha induced apoptosis of MI |
| k52 | 0.6 | 0.7 | 1/day | [1], [2] | Cytotoxic killing of MI |
| w1 | 0.2 | 0.7 | -- | [1], [2], fit | Max contribution of Th1 to cytotoxic killing |
| c52 | 103290 | 246770 | -- | fit | Half saturation of TC on MI killing |
| cT1 | 35 | 35 | -- | fit | Half saturation of Th1 on cytotoxic killing |
| nuMI | 0.0033 | 0.0033 | 1/day | [3], [2], [5] | MI death rate |
| nuMA | 0.17 | 0.17 | 1/day | [3], [2], fit | MA death rate |
| alpha1a | 0.03 | 0.55 | 1/day | [6], [2], fit | Macrophage recruitment of T0 |
| Sr1b | 2E+04 | 5E+4 | 1/day | [6], [2], fit | Falpha dependent T0 recruitment |
| alpha2 | 0.12 | 0.36 | 1/day | [3], [2], fit | Max growth rate of T0 |
| c15 | 2.75E+06 | 4.09E+06 | -- | [3], [2], fit | Half saturation of MA on IFNg production by Th1 |
| k6 | 0.1 | 0.2 | ml/(pg day) | [3], [2], fit | Max T0 to Th1 rate |
| f7 | 7 | 30 | -- | [1], [2], fit | Effect of IL-10 on IFNg induced differentiation of T0 to Th1 |
| k7 | 0.25 | 0.64 | ml/(pg day) | [3], [2], fit | Max T0 to Th2 rate |
| f2 | 0.2 | 0.4 | -- | fit | Adjustment IFNg/IL-4 |
| s2 | 400 | 900 | pg/day | fit | Half saturation IL-4 |
| nuT0 | 0.22 | 0.22 | 1/day | [3], [2], fit | T0 death rate |
| CD8MultiFunc | 0.7 | 0.9 | -- | [1], [2], fit | overlap between TC and T8 function |
| alpha3a | 0.4 | 0.8 | 1/day | fit | Macrophage recruitment of Th1 |
| Sr3b | 15 | 80 | 1/day | Fit | Falpha dependent recruitment of Th2 |
| alpha3a2 | 0.22 | 0.75 | 1/day | fit | Macrophage recruitment of Th2 |
| Sr3b2 | 50 | 90 | 1/day | fit | Falpha dependent recruitment of Th2 |
| nuTg | 0.24 | 0.75 | 1/day | fit | IFNg induced apoptosis of Th1 |
| c | 270 | 690 | pg/ml | fit | Half saturation IFNg on Th1 apoptosis |
| nuT1 | 0.33 | 0.33 | 1/day | [3], [2] | Th1 death rate |
| nuT2 | 0.33 | 0.33 | 1/day | [3], [2] | Th2 death rate |
| alpha3ac | 0.25 | 0.77 | 1/day | fit | Macrophage recruitment of TC and T8 |
| Sr3bc | 14 | 26 | 1/day | fit | Falpha dependent recruitment of TC and T8 |
| nuTCg | 0.45 | 0.83 | 1/day | fit | IFNg induced apoptosis of TC and T8 |
| cc | 350 | 590 | pg/ml | [7], [2], fit | Half saturation of IL on TC and T8 apoptosis |
| nuTC | 0.3 | 0.3 | 1/day | [1] | TC death rate |
| sg | 2375 | 7340 | pg/(ml day) | fit | IFNg production by cells |
| c10 | 5.50E+05 | 6.35E+06 | count | [3], [2], fit | Half saturation of Mtb on IFNg production by cells |
| s7 | 590 | 820 | pg/ml | fit | Half saturation of IL-12 on IFNg production by cells |
| alpha5a | 0.6 | 0.8 | pg/day | [1], [2], fit | IFNg production by Th1 |
| c5a | 315 | 630 | 1/ml | fit | Half saturation of MA on IFNg production by Th1 |
| alpha5b | 0.15 | 0.58 | pg/day | [1], [2], fit | IFNg production by T8 |
| alpha5c | 0.08 | 0.35 | pg/ml | [1], [2], fit | IFNg production by MI |
| c5b | 160 | 795 | count | fit | Half saturation of MA on IFNg production by T8 |
| alpha7 | 0.012 | 0.16 | pg/ml | [3], [2], fit | IFNg production by T0 |
| f4 | 1.5 | 1.5 | -- | [3], [2], fit | Adjustment of IL-10/IL-12 on IFNg |
| s4 | 270 | 890 | pg/ml | [3], [2], fit | Half saturation of IL-12 on IFNg |
| nuIG | 6 | 9 | 1/day | [3], [2], fit | IFNg decay rate |
| alpha23 | 0.004 | 0.004 | pg/ml | [1], [2], fit | IL-12 production by MR |
| c23 | 140 | 525 | 1/ml | [1], [2], fit | Half saturation of Mtb on IL-12 production by MR |
| alpha8 | 0.38 | 0.80 | pg/day | [3], [2], fit | IL-12 Production by MA |
| s12 | 2330 | 3650 | pg/(ml day) | [1], [2], fit | Cell production of IL-12 |
| c230 | 390 | 710 | count | Fit | Half saturation of Mtb on IL-12 production by DC’s |
| nuIL-12 | 1.1 | 1.1 | 1/day | [3] | IL-12 death rate |
| s | 170 | 650 | pg/ml | fit | IL-10 effect on IL-12 production by MA |
| s6 | 680 | 770 | pg/ml | Fit | Half saturation of IL-10 self-inhibition in MA |
| f6 | 0.35 | 0.35 | -- | [3] | Adjustment IFNg on IL-10 |
| delta7 | 0.40 | 0.8 | pg/ml | fit | IL-10 production by MA |
| alpha16 | 0.33 | 0.8 | pg/day | Fit, [5] | IL-10 production by Th1 |
| alpha17 | 0.3 | 0.5 | pg/day | Fit, [5] | IL-10 production by Th2 |
| alpha18 | 0.5 | 0.7 | pg/day | Fit, [5] | IL-10 production by TC and T8 |
| nuIL-10 | 1.81 | 4.1 | 1/day | [3], fit | IL-10 decay rate |
| alpha11 | 0.0033 | 0.073 | pg/day | [3], fit | IL-4 production by T0 |
| alpha12 | 0.02 | 0.06 | pg/day | [3], fit | IL-4 production by Th2 |
| nuIL-4 | 2.7 | 2.7 | 1/day | [3] | IL-4 decay rate |
| alpha30 | 0.05 | 0.09 | pg/(ml day) | [1], fit | Falpha production by MI |
| alpha31 | 0.15 | 0.78 | pg/(ml day) | [1], fit | Falpha production by MA |
| beta2 | 12000 | 12000 | 1/pg | [1], fit | Scaling factor of Mtb for Falpha production by MA |
| s10 | 100 | 300 | pg/ml | [1], fit | Half saturation of IFNg on Falpha production by MA |
| alpha32 | 0.2 | 0.3 | pg/(ml day) | fit | Falpha production by Th1 |
| alpha33 | 0.2 | 0.3 | pg/(ml day) | Fit | Falpha production by T8 |
| nuTNF | 1.1 | 1.1 | 1/day | [8] | Falpha decay rate |
| alpha19 | 0.87 | 1.27 | 1/day | [3], fit | BI replication rate |
| alpha20 | 0.3 | 0.4 | 1/day | [3], fit | BE replication rate |
| Nfracc | 0.06 | 0.06 | -- | [3] | Fraction BI released by T cell apoptosis of MI |
| Nfraca | 0.06 | 0.06 | -- | [3] | Fraction BI released by TNF apoptosis of MI |
| k15 | 0.0002 | 0.001 | 1/day | [3], fit | BE killing by MA |
| k18 | 0.0001 | 0.0007 | 1/day | [3], fit | BE killing by MR |
| nI | 6.3E-05 | 8.3E-05 | 1/day | [1], fit | BI death rate |
| nE | 4.4E-09 | 6.65E-09 | 1/day | [1], fit | BE death rate |
| Nfracd | 0.001 | 0.001 | -- | fit | Fraction of BI released by natural death of MI |

**Table S2: ODE model parameters that govern individual granuloma formation and growth across time.**

*For each disseminating granuloma, we allow for the option to sample each parameter from a subrange smaller than its parent’s ranges. We do this by using a fraction between 0 and 1 (inclusive) to determine the limits of the range. The fraction represents the percent of values between the parent’s value and either extrema (minimum and maximum) to include in the range. 0 means the range includes only the parent’s value; 1 means that the original range is used.

1. Sud D, Bigbee C, Flynn JL, Kirschner DE. Contribution of CD8+ T Cells to Control of Mycobacterium tuberculosis Infection. J Immunol. 2014;176: 4296–4314. doi:10.4049/jimmunol.176.7.4296

2. Guzzetta G, Kirschner D. The Roles of Immune Memory and Aging in Protective Immunity and Endogenous Reactivation of Tuberculosis. PLoS One. 2013;8. doi:10.1371/journal.pone.0060425

3. Wigginton JE, Kirschner D. A Model to Predict Cell-Mediated Immune Regulatory Mechanisms During Human Infection with Mycobacterium tuberculosis. J Immunol. 2001;166: 1951–1967. doi:10.4049/jimmunol.166.3.1951

4. Gilbertson B, Zhong J, Cheers C. Anergy, IFN-gamma production, and apoptosis in terminal infection of mice with Mycobacterium avium. J Immunol. 1999;163: 2073–2080.

5. Cilfone NA, Ford CB, Marino S, Mattila JT, Gideon HP, Flynn JL, et al. Computational Modeling Predicts IL-10 Control of Lesion Sterilization by Balancing Early Host Immunity–Mediated Antimicrobial Responses with Caseation during Mycobacterium tuberculosis Infection . J Immunol. 2015;194: 664–677. doi:10.4049/jimmunol.1400734

6. Tsukaguchi K, De Lange B, Boom WH. Differential regulation of IFN-γ, TNF-α, and IL-10 production by CD4+ αβTCR+ T cells and Vδ2+ γδ T cells in response to monocytes infected with Mycobacterium tuberculosis-H37Ra. Cell Immunol. 1999;194: 12–20. doi:10.1006/cimm.1999.1497

7. Oddo M, Renno T, Attinger A, Bakker T, MacDonald HR, Meylan PR. Fas ligand-induced apoptosis of infected human macrophages reduces the viability of intracellular Mycobacterium tuberculosis. J Immunol. 1998;160: 5448–54. Available: http://www.ncbi.nlm.nih.gov/pubmed/9605147

8. D’Amico G, Frascaroli G, Bianchi G, Transidico P, Doni A, Vecchi A, et al. Uncoupling of inflammatory chemokine receptors by IL-10: Generation of functional decoys. Nat Immunol. 2000;1: 387–391. doi:10.1038/80819
