# Supplementary material for: A computational model tracks whole-lung Mycobacterium tuberculosis infection and predicts factors that inhibit dissemination
Source: PLoS Comput Biol. 2020 May 20;16(5):e1007280. doi: 10.1371/journal.pcbi.1007280 (PMC7239387; doi:10.1371/journal.pcbi.1007280)
Supplement: S2 Table — (DOCX) [file pcbi.1007280.s003.docx]

| Parameter name | Value | Units | Ref | Description |
| --- | --- | --- | --- | --- |
| diamMacs | 20 | microns | [40] | Diameter of Macrophage |
| diamTCells | 5 | microns | [40] | Diameter of T cell |
| dt | 1 | day | ~ | Agent time step |

**Table S3: Other parameters for size of granulomas and runtime execution.**
