# Supplementary material for: A computational model tracks whole-lung Mycobacterium tuberculosis infection and predicts factors that inhibit dissemination
Source: PLoS Comput Biol. 2020 May 20;16(5):e1007280. doi: 10.1371/journal.pcbi.1007280 (PMC7239387; doi:10.1371/journal.pcbi.1007280)
Supplement: S3 Table — (DOCX) [file pcbi.1007280.s004.docx]

| Parameter name | Min | Max | Units | Reference | Description |
| --- | --- | --- | --- | --- | --- |
| DissemDistMean | ${10}^{0}$ | ${10}^{1}$ | um | Fit, Based on data [1] | Mean distance of local dissemination |
| Lambda_Local | ${10}^{-3}$ | ${10}^{-1}$ | CFU/sec | Fit, Based on data [1] | Max probability of local dissemination |
| CFU_Half_Local | ${10}^{3}$ | ${10}^{4}$ | CFU | Fit, Based on data [1] | Value for half of max rate of local dissemination |
| Lambda_NonLocal | ${10}^{-3.5}$ | ${10}^{-1.5}$ | CFU/sec | Fit, Based on data [1] | Max probability of non-local dissemination |
| CFU_Half_NonLocal | ${10}^{3.5}$ | ${10}^{4.5}$ | CFU | Fit, Based on data [1] | Value for half of max rate of non-local dissemination |
| TcellFracDonateMu | 1/100 | 1/10 | -- | estimated | Mean fraction of all of the parent granuloma’s T cells that move to the daughter granuloma during a local dissemination event |
| TcellFracDonateSig | ${10}^{-3}$ | ${10}^{-2}$ | -- | estimated | Standard deviation from the mean fraction of all of the parent granuloma’s Tcells that move to the daughter granuloma during a local dissemination event |

**Table S4: Dissemination Parameters.** These seven parameters dictate dissemination dynamics in *MultiGran*. Parameters were fit to barcode data or varied using Uncertainty Analysis to find an estimation.

1. Martin CJ, Cadena AM, Leung VW, Lin PL, Maiello P, Hicks N, et al. Digitally Barcoding Mycobacterium tuberculosis Reveals In Vivo Infection Dynamics in the Macaque Model of Tuberculosis . MBio. 2017;8. doi:10.1128/mbio.00312-17
