# Supplementary figures and images for: A computational model tracks whole-lung Mycobacterium tuberculosis infection and predicts factors that inhibit dissemination
Source: PLoS Comput Biol. 2020 May 20;16(5):e1007280. doi: 10.1371/journal.pcbi.1007280 (PMC7239387; doi:10.1371/journal.pcbi.1007280)

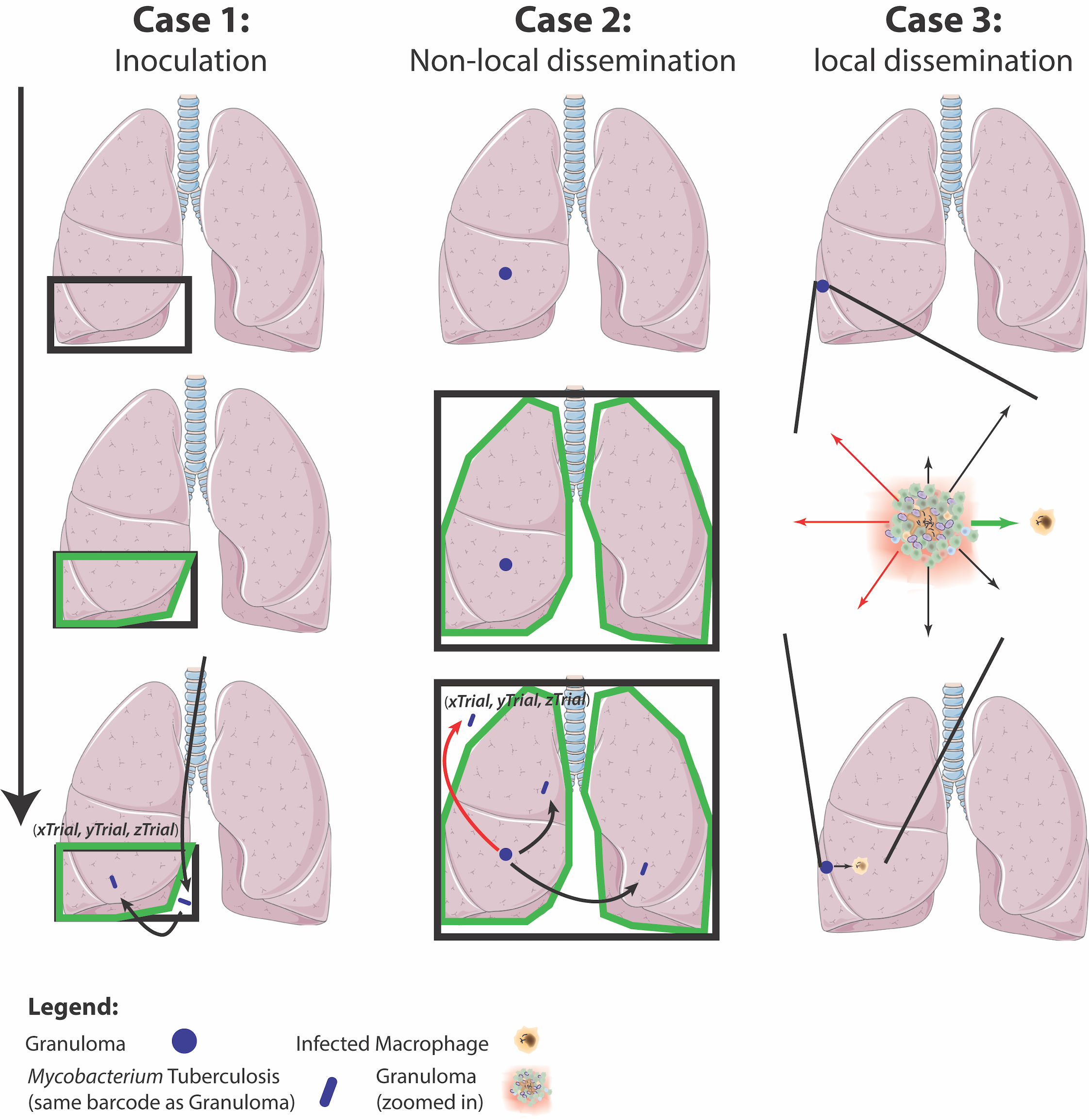

Supplement: S1 Fig — Inoculation deposits bacteria in a specific lung region at position (xTrial, yTrail, zTrial). The black box designates inoculation region (row 1), wherein the specific within-lung region destined for inoculation is highlighted in green (row 2). The third row demonstrates successful inoculation of a single bacterium–the black box was sampled randomly until the sampled coordinates lie within the green region. Cases 2 and 3 define granuloma placement following dissemination. Case2 –non-local dissemination. When non-local dissemination occurs, a bacterium escapes a single granuloma (row 1) and can be placed in any region (shown in black in row 2) that encompasses the entire lung. The green highlighted region is the area in which the bacterial placement will be accepted. Row 3 shows three trial placements: two realizations of accepted bacterial placement (black arrows) and one unaccepted placement (red arrow) at (xTrial, yTrial, zTrial). Case 3– local dissemination. Local dissemination is the only form of granuloma placement which does not utilize random placement within a region of lung space. Rather, an infected macrophage from the parent granuloma is placed in a random direction away from the parent granuloma. Row 2 shows several options for granuloma infected macrophage placement. Note that the arrows are of different length to represent our assumption that local dissemination likely follows a normal distribution with respect to parent granuloma location. Here, the green and black arrows show valid directions for the new placement for the infected macrophage, while red arrows show invalid directions. A new granuloma will begin to develop in the chosen (green) valid location (Row 3). Note that in both (A) and (B) bacteria, granulomas, and infected macrophages are not to scale. Lung image from Servier Medical Art. (TIF) [file pcbi.1007280.s005.tif]
